# Multimorbidity patterns and mortality in older adults: Results from the KORA-Age study

**DOI:** 10.3389/fnut.2023.1146442

**Published:** 2023-03-27

**Authors:** Ava Arshadipour, Barbara Thorand, Birgit Linkohr, Karl-Heinz Ladwig, Margit Heier, Annette Peters

**Affiliations:** ^1^Institute of Epidemiology, Helmholtz Zentrum München, German Research Center for Environmental Health, Munich, Germany; ^2^Institute for Medical Information Processing Biometry and Epidemiology (IBE), Ludwig-Maximilians-Universität München, Munich, Germany; ^3^German Center for Diabetes Research (DZD), Neuherberg, Germany; ^4^Department for Psychosomatic Medicine and Psychotherapy, Klinikum Rechts Der Isar, Technical University of München, Munich, Germany; ^5^KORA Study Centre, University Hospital of Augsburg, Augsburg, Germany; ^6^German Center for Cardiovascular Disease Research (DZHK), Munich, Germany

**Keywords:** chronic disease, multimorbidity, mortality, older people, sex differences, hazard ratios

## Abstract

The coexistence of several chronic diseases is very common in older adults, making it crucial to understand multimorbidity (MM) patterns and associated mortality. We aimed to determine the prevalence of MM and common chronic disease combinations, as well as their impact on mortality in men and women aged 65 years and older using the population-based KORA-Age study, based in South of Germany. The chronic disease status of the participants was determined in 2008/9, and mortality status was followed up until 2016. MM was defined as having at least two chronic diseases. We used Cox proportional hazard models to calculate the hazard ratios (HRs) and the 95% confidence intervals (CIs) for associations between MM and all-cause mortality. During the study period 495 men (24.6%) and 368 women (17.4%) died. Although the MM prevalence was almost the same in men (57.7%) and women (60.0%), the overall effect of MM on mortality was higher in men (HR: 1.81, 95% CI: 1.47–2.24) than in women (HR: 1.28, 95% CI: 1.01–1.64; *p*-value for interaction <0.001). The type of disease included in the MM patterns had a significant impact on mortality risk. For example, when both heart disease and diabetes were included in the combinations of two and three diseases, the mortality risk was highest. The risk of premature death does not only depend on the number of diseases but also on the specific disease combinations. In this study, life expectancy depended strongly on a few diseases, such as diabetes, hypertension, and heart disease.

## 1. Introduction

Even though life expectancy has increased in recent decades as a result of modern medicine, individuals are developing more chronic diseases, resulting in rising multimorbidity (MM) (1). According to the World Health Organization (WHO), MM has been defined as the occurrence of two or more chronic diseases in one person at the same time (2).

Based on a systematic literature review of 41 articles from different countries, the prevalence of MM ranges from 55 to 98% in those aged ≥65 years. In Germany, based on the cross-sectional national telephone health interview survey “German Health Update” (GEDA 2012–2013), the MM prevalence ranged from 61.7% (95% CI: 59.3–64.1) for 60 to 69-year-olds to 72.9% (95% CI: 70.4–75.2) for 70 to 79-year-old individuals. Others reported a 62% MM prevalence for those aged ≥65 years in the German population. In Augsburg, MM prevalence was 58.6% for individuals aged 65–94 years based on the KORA-Age data in 2008/9 (3).

Investigating chronic illness combinations and their negative consequences on the old people and the health care system is a major concern in countries with growing aging populations nowadays. Many studies showed the association between MM and lower quality of life (4), higher health care use and cost (5), and functional decline in older adults (6).

Moreover, many studies have found a relationship between MM and an increased risk of mortality (7–12), but the value of mortality risk is not similar. One reason for these dissimilarities could be a low sample size in some studies, and another reason for the differences might be the included disease types, age groups, and risk factors (7–12).

To our knowledge, there exists only one recent study (13) in Germany exploring the association of MM patterns and mortality based on health insurance claims data. Therefore, we aimed to identify the prospective association of MM with all-cause mortality controlling for sociodemographic and lifestyle factors in men and women based on the large population-based KORA-Age study. Additionally, we specified the most prevalent combinations of disease in men and women, and their associated mortality risk.

## 2. Methods

### 2.1. Data collection and study design

The adult population-based KORA (Cooperative Health Research in the Region of Augsburg) study was conducted between 1984 and 2001 in the Region of Augsburg, Germany. In 2008/9, KORA participants who were aged 65-year-olds or older were invited for the first wave of a specific project on health in old age – the KORA-Age study. Details about the study design and data collection have been explained elsewhere (14). Briefly, 5.991 individuals from the KORA cohort who were still alive, had not moved outside the study area or had not withdrawn their consent to participate met the inclusion criteria born between 1915 and before 1944 (i.e., ≥65 years in 2009). 4,565 individuals returned a postal self-report questionnaire and 4,127 individuals (2015 men and 2,112 women) answered further questions in a 30 min standardized telephone interview. The questionnaire and interview items are based the MONICA (Monitoring Trends and Determinants in Cardiovascular Disease) Project of the World Health Organization or from validated instruments. For instance, the MM instruments chosen were from established questionnaires (15–17). The study was conducted by trained staff at the KORA study Center in Augsburg after an initial pilot phase. Mortality status was assessed until 2016 by official death certificates. 4,127 individuals with questionnaire and interview information were included in this analysis.

### 2.2. Mortality

All participants of the first wave of the KORA-Age study were followed for all-cause and cause-specific (cardiovascular, cancer, and other disease-related) mortality using official death certificates, coded according to the International Classification of Diseases (ICD-10). Cardiovascular-related mortality consists of diseases of the circulatory system (ICD-9 codes 390–459, ICD-10 codes I00–I99) and sudden death with unknown cause (ICD-9 code 798, ICD-10 code R99). Cancer-related mortality consists of neoplasms (ICD-9 codes 140–208, ICD-10 codes C00–C95). Other disease-related mortality consists of the remaining causes of death, for example, pneumonia (ICD-9 code 486, ICD-10 codes J18.8, J18.9), chronic bronchitis (ICD-9 code 491, ICD-10 Codes J41, J42, J44) and dementias (ICD-9 code 290, ICD-10 Codes F03.90, F05, F01.50, F01.51) (18). Follow-up for participant’s mortality status was performed until 07.10.2016 (median follow-up time: 6.97 years, interquartile range; IQR = 75th%–25th%: 7.07–6.74 years).

### 2.3. Multimorbidity and single chronic diseases

MM has been defined as the presence of two or more chronic diseases in one person simultaneously (2). We considered 14 major chronic diseases, including hypertension, eye disease, heart disease, diabetes, joint disease, lung disease, gastrointestinal disease, stroke, cancer, kidney diseases, liver diseases, neurological diseases, depression, and anxiety. Hypertension, diabetes, cancer (any cancer recognized within the last 3 years), stroke, and heart diseases (myocardial infarction and coronary artery disease) were assessed based on the self-report questionnaire (whether participant currently have disease). All other diseases were identified in a telephone interview based on the Charlson Comorbidity Index (15). Participants were asked whether they suffer from kidney, liver, lung diseases (e.g., asthma, chronic bronchitis, and emphysema), inflammatory joint problems (arthritis or rheumatism), gastrointestinal diseases (e.g., colitis, cholecystic, gastric, or ulcer), heart diseases (e.g., congestive heart failure, coronary heart failure, or angina), eye problems (e.g., cataract, retinitis pigmentosa, glaucoma, macular degeneration, diabetic retinopathy). Neurological diseases were evaluated based on self-reported diseases like epilepsy, Parkinson’s disease, or multiple sclerosis. The Geriatric Depression Scale (16) and Generalized Anxiety Disorder Scale-7 (17) screening tools were used to diagnose depression and anxiety. Persons with scores >10 were defined as suffering from depression or anxiety.

### 2.4. Demographic and lifestyle measures

We considered age, family status, education level, alcohol consumption, physical activity, body mass index (BMI), and smoking behavior as covariates. Family status is a combination of the self-reported marital status and living alone or with a spouse/partner categorized in the two groups “living with a partner/spouse” and “living alone, divorced or widowed.” The education level had three categories based on years of education and vocational training: low level (9 years or less), middle (10 or 11 years), and high (12 years or more).

Alcohol consumption was based on self-reported alcohol intake with the following three groups: “Never, rare or former use,” “once a week,” or “daily use.” Leisure time physical activity was measured from two separate questions about leisure time sports activity per week in winter and summer, including cycling. Possible answers were (1) >2 h, (2) 1–2 h, (3) <1 h, and (4) none. Participants, who had a total score of <5, obtained by summing the numbers (1)–(4) relating to activities in winter and summer, were classified to be “physically active” (19).

The BMI was computed by dividing weight in kilograms by square height in meters. Measurements of height and weight were made by trained investigators while wearing light clothes and without shoes. Based on self-reported information, there are three categories for smoking status: never smokers, former smokers, and active smokers.

### 2.5. Statistical analysis

We presented categorical data as percentages and continuous data as means (SD) if they were normally distributed or medians (IQR) if non-normally distributed in the descriptive analysis. To examine the differences between outcome groups (alive and dead), *t*-test for continuous variables and the Chi-squared test for categorical variables were performed. Kaplan–Meier curves and log-rank tests were presented graphically to compare the survival distributions of participants with and without MM.

Cox proportional-hazards models were used to investigate the associations between MM and specific combinations of disease with mortality by adjusting for age, education, family status, smoking habits, alcohol use, BMI, and physical activity. The combined model has been used to check the significance of sex differences and then the sex-specific models have been performed. The interaction effect of MM with age and BMI was also checked in the sex specific models. Moreover, the spline Cox proportional hazard model was used for examining the non-linear effect of BMI. The proportional hazard assumption was examined using Schoenfeld residuals. Additionally, the prevalence of every single disease in men and women was calculated. In order to check the patterns of disease combinations, all possible combinations of two and three diseases were identified and the most prevalent combinations were presented in men and women separately. Using those with no disease or just one disease as the reference group, the adjusted hazard ratios of these most common combinations were then calculated.

For sensitivity analysis, we adjusted the model examining the association between MM and mortality for waist-to-hip ratio instead of BMI. Additionally, we fitted the model without MM to check to which degree MM can explain the underlying association between risk factors and mortality. We also ran the model without BMI to evaluate how much BMI could confound the effect of MM on mortality. Statistical relationships were considered significant for *p*-values <0.05. All statistical analyses were performed using R 4.1.2 and RStudio 2021.09.1^1^ and the “dplyr,” “pspline,” “survival” and “survminer” libraries for analysis has been used.

### 2.6. Ethics statement

The Ethics Committee of the Bavarian Medical Association has approved the KORA-Age study (08094). Written informed consent was obtained from all study participants according to the Helsinki Declaration.

## 3. Results

### 3.1. Study population characteristics

**Figure 1:**
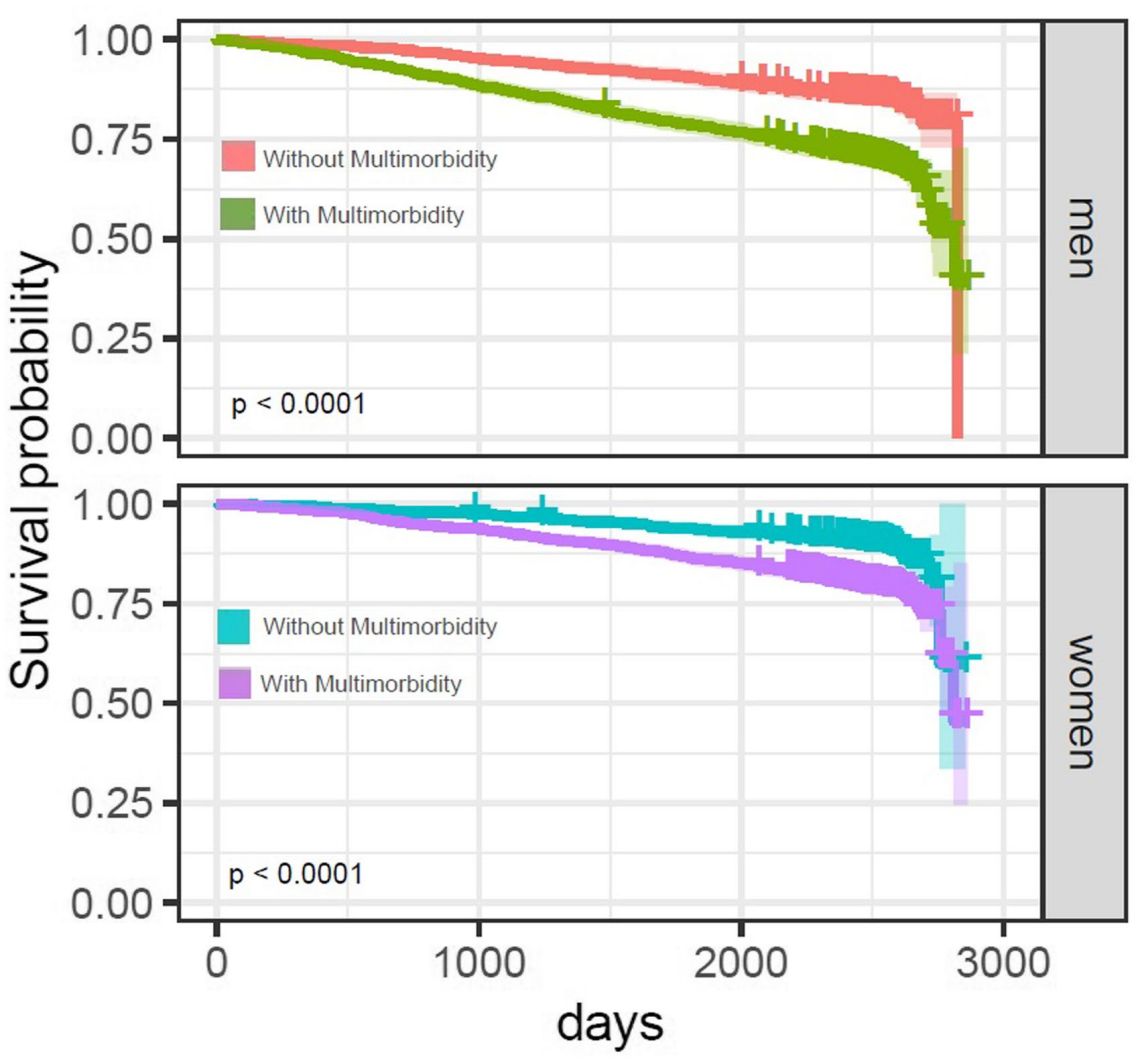
Kaplan–Meier survival curves for men and women with and without multimorbidity. Kaplan–Meier curve shows the time to death for individuals with and without MM. Censored data (vertical sign-on lines) denotes participants no longer available in KORA-Age 3 who did not experience death. There were statistically significant (*p* < 0.0001) differences in survival probability between 2 groups in both men and women based on the log-rank test.

The prevalence of MM was 57.7 and 60.0% in men and women, respectively. Baseline characteristics of the 4,127 participants stratified by their mortality status are shown in Table 1. Out of 2015 male participants, 495 (24.5%) died (24.1% cancer related, 44.5% CVD-related and 31.4% other disease-related deaths). Out of 2,112 female participants, 368 (17.5%) died (23.1% cancer-related, 44.8% CVD-related, and 32.1% other disease-related deaths). There were statistically significant differences (*p* < 0.001) between those who survived and those who died for age, family status, education, alcohol use, physical activity, and MM status in both men and women (Table 1). Although associations of BMI and smoking habits with mortality were statistically significant in men, they were not significant in women. Individuals without MM had a significantly longer survival probability compared to those with MM in both men and women (Figure 1).

**Table 1:** Baseline characteristics of the study participants were stratified by sex and mortality status.

Characteristics		Men			Women		
		Died = No	Died = Yes	Value of *p*	Died = No	Died = Yes	Value of *p*
*N*		1,520	495	…	1744	368	…
Age [mean (SD)]		71.85 (5.34)	77.45 (6.20)	< 0.001	72.3 (5.39)	78.3 (6.66)	<0.001
Multimorbidity (%)	Yes	799 (52.6)	364 (73.5)	< 0.001	993 (56.9)	274 (74.5)	<0.001
	No	721 (47.4)	131 (26.5)		751 (43.1)	94 (25.5)	
Family status (%)	Living alone, divorced, widowed	262 (17.2)	144 (29.1)	< 0.001	965 (55.3)	118 (32.1)	<0.001
	Living with a partner/spouse	1,258 (82.8)	351 (70.9)		779 (44.7)	250 (67.9)	
Education (%)	Low (8–9 years)	85 (5.6)	52 (10.5)	< 0.001	466 (26.7)	138 (37.5)	< 0.001
	Medium (10–11 years)	800 (52.6)	292 (59.0)		1,012 (58.0)	182 (49.5)	
	High (12 or higher years)	635 (41.8)	151 (30.5)		266 (15.3)	48 (13.0)	
Alcohol (%)	Never, rare or former use	330 (21.7)	142 (28.7)	0.006	977 (56.0)	239 (64.9)	0.004
	Once a week	192 (12.6)	56 (11.3)		251 (14.4)	36.0 (9.8)	
	Daily use	998 (65.7)	297 (60.0)		516 (29.6)	93 (25.3)	
BMI-kg/m^2^ (SD)		27.67 (3.59)	27.13 (3.81)	0.004	27.35 (4.55)	26.94 (4.98)	0.122
Smoking (%)	Never Smoker	518 (34.1)	128 (25.9)	0.003	1,231 (70.6)	262 (71.6)	0.637
	Former smoker	900 (59.3)	330 (66.7)		414 (23.7)	80 (21.9)	
	Current smoker	100 (6.6)	37 (7.5)		99 (5.7)	24 (6.6)	
Physical activity (%)	Active	993 (65.3)	201 (39.9)	< 0.001	992 (56.9)	113 (30.3)	<0.001
	Inactive	527 (34.7)	294 (60.1)		752 (43.1)	255 (69.7)	

Arithmetic means (SD) were given for age and BMI as continuous variables and frequency (percentage) for other categorical variables. *T*-test for continuous variables and the chi-squared test for categorical variables were used to check the difference between groups.

### 3.2. Association between multimorbidity and all-cause mortality

Based on a non-linear multivariable-adjusted model, MM status was significantly positively associated with all-cause mortality in men (HR: 1.81, 95% CI: 1.47–2.24) and women (HR: 1.28, 95% CI: 1.01–1.64; Table 2).

**Table 2:** Multivariate adjusted association of MM and all-cause mortality for men and women.

Characteristics		Men (*n* = 2015)				Women (*n* = 2,112)			
		Model 0	Model 1	Model 2	Model 3 (non-linear BMI)	Model 0	Model 1	Model 2	Model 3 (non-linear BMI)
Multimorbidity (ref: no)		2.32 (1.89,2.85)	1.84 (1.51,2.26)	1.82 (1.48,2.24)	1.81 (1.47, 2.24)	2.10 (1.66,2.66)	1.35 (1.05,1.72)	1.29 (1.01,1.65)	1.28 (1.01,1.64)
Age (per year)			1.13 (1.12,1.15)	1.12 (1.09,1.13)	1.11 (1.09, 1.12)		1.15 (1.13,1.17)	1.12 (1.10,1.15)	1.12 (1.11,1.15)
Family status (ref: Living with a partner/spouse)	Living alone, divorced, widowed	…	…	1.36 (1.12,1.67)	1.35 (1.11,1.66)	…	…	1.27 (0.99,1.61)	1.25 (0.98,1.59)
Education (ref: 12 years or more)	8–9 years	…		1.50 (1.09,2.07)	1.49 (1.08,2.06)	…		1.15 (0.82,1.62)	1.20 (0.85,1.69)
	10–11 years			1.29 (1.05,1.57)	1.30 (1.06,1.59)			0.93 (0.67,1.28)	0.96 (0.69,1.33)
Alcohol use (ref: once a week)	Never, rare or former use	…		1.29 (1.09,2.07)	1.18 (0.85,1.64)	…		1.25 (0.87,1.79)	1.21 (0.84,1.74)
	Daily use			1.04 (0.77,1.41)	1.04 (0.76,1.40)			1.11 (0.75,1.65)	1.08 (0.73,1.61)
Physical activity (ref: active)		…		1.70 (1.40,2.04)	1.68 (1.39,2.00)	…		1.70 (1.35,2.15)	1.67 (1.32,2.11)
BMI (kg/m^2^)		…		0.97 (0.94,0.99)	*	…		0.99 (0.97,1.01)	*
Smoking status (ref: never)	Former smoker	…		1.16 (0.94,1.43)	1.15 (0.93,1.42)	…		1.08 (0.83,1.39)	1.04 (0.880,1.35)
	Current smoker			1.76 (1.21,2.56)	1.73 (1.18,2.51)			1.59 (1.03,2.46)	1.54 (1.00,2.39)
AIC		7070.243	6794.823	6740.871	6730.415	5305.573	5050.834	5032.326	5026.419

Data were presented as hazard ratios with 95% confidence intervals. Model 0, model 1, and model 2 were calculated based on the Cox proportional hazard model.

* In model 3, BMI was considered as the spline effect in the spline Cox proportional hazard model.

### 3.3. Risk factor profiles of all-cause mortality

Age, family status, educational attainment, physical activity, and smoking were significantly linked to increased mortality risk in males, whereas age, physical activity, and smoking were significantly linked to increased mortality risk in women (Table 2).

Since the interaction effect between age and MM was significant, we stratified our analysis by 5-year age groups.

**Figure 2:**
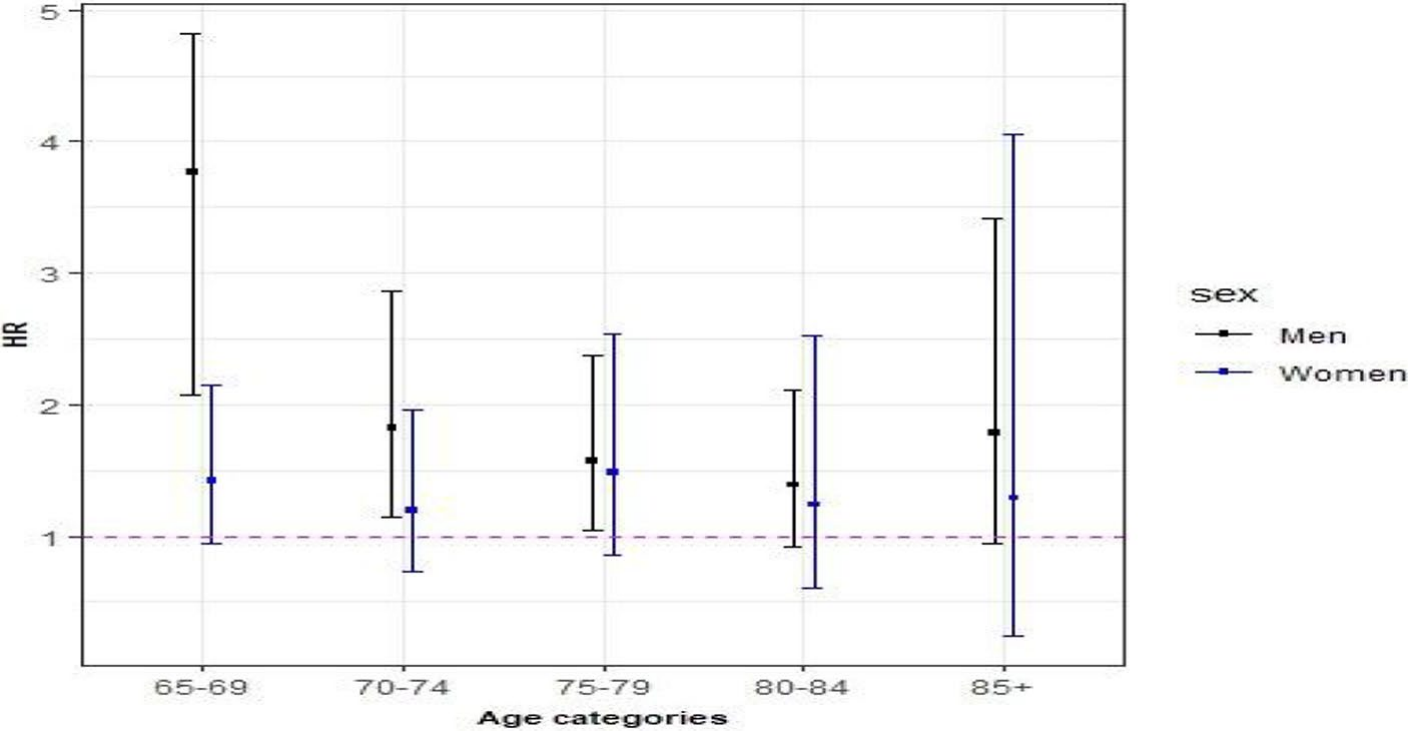
Age stratified hazard ratios for the association of multimorbidity with all-cause mortality for men and women. Hazard ratios (95% CIs) for associations of MM with all-cause mortality were calculated for each age group and adjusted for education, family status, smoking habits, alcohol use, BMI, and physical activity.

**Figure 3:**
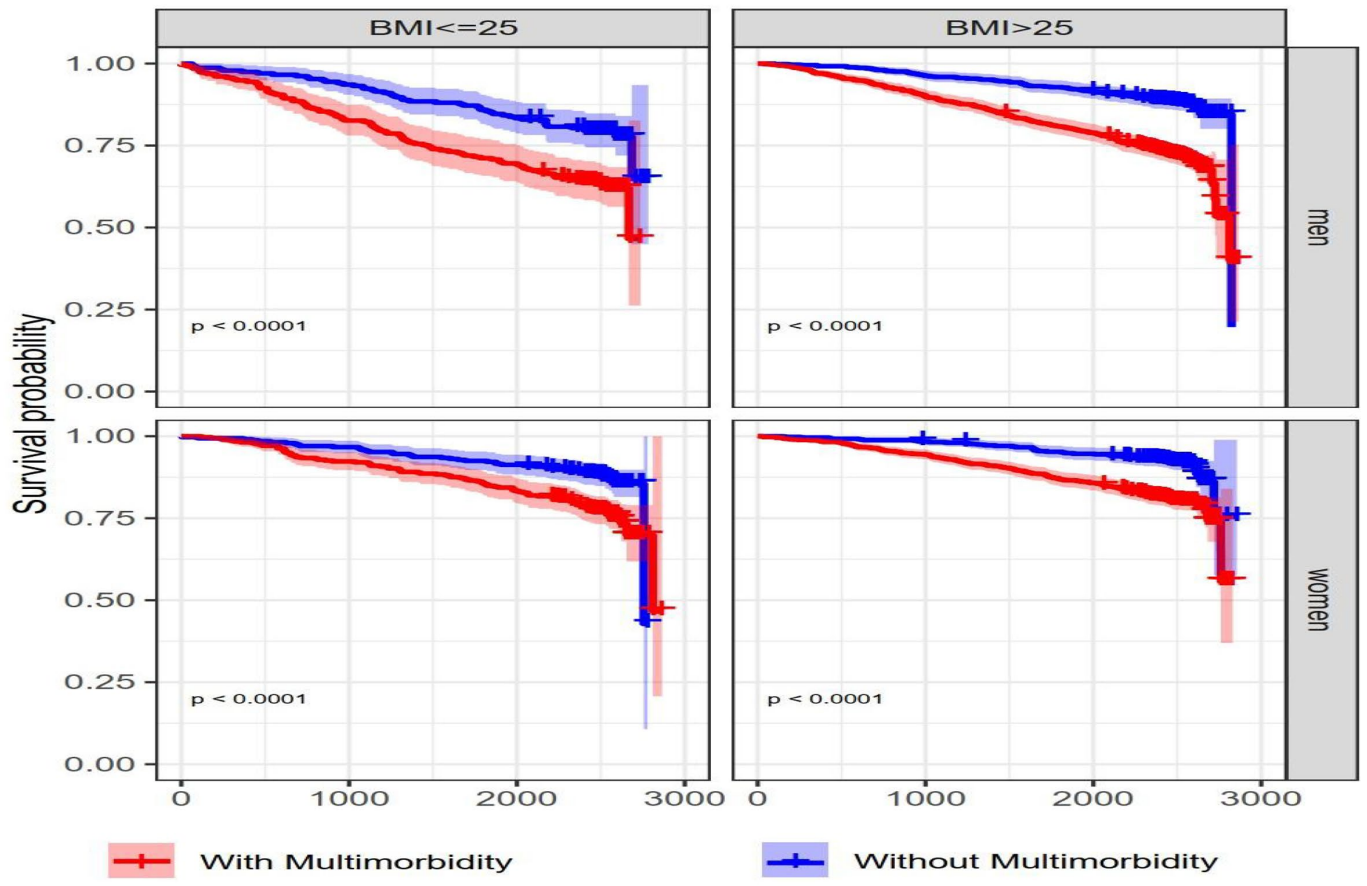
Kaplan–Meier survival curves for men and women based on BMI and multimorbidity status. Kaplan–Meier curves show the comparison of survival probability between individuals with and without MM stratified by BMI category (<=25, >25) and sex. *p*-value <0.0001 are presented for the log-rank test.

**Figure 4:**
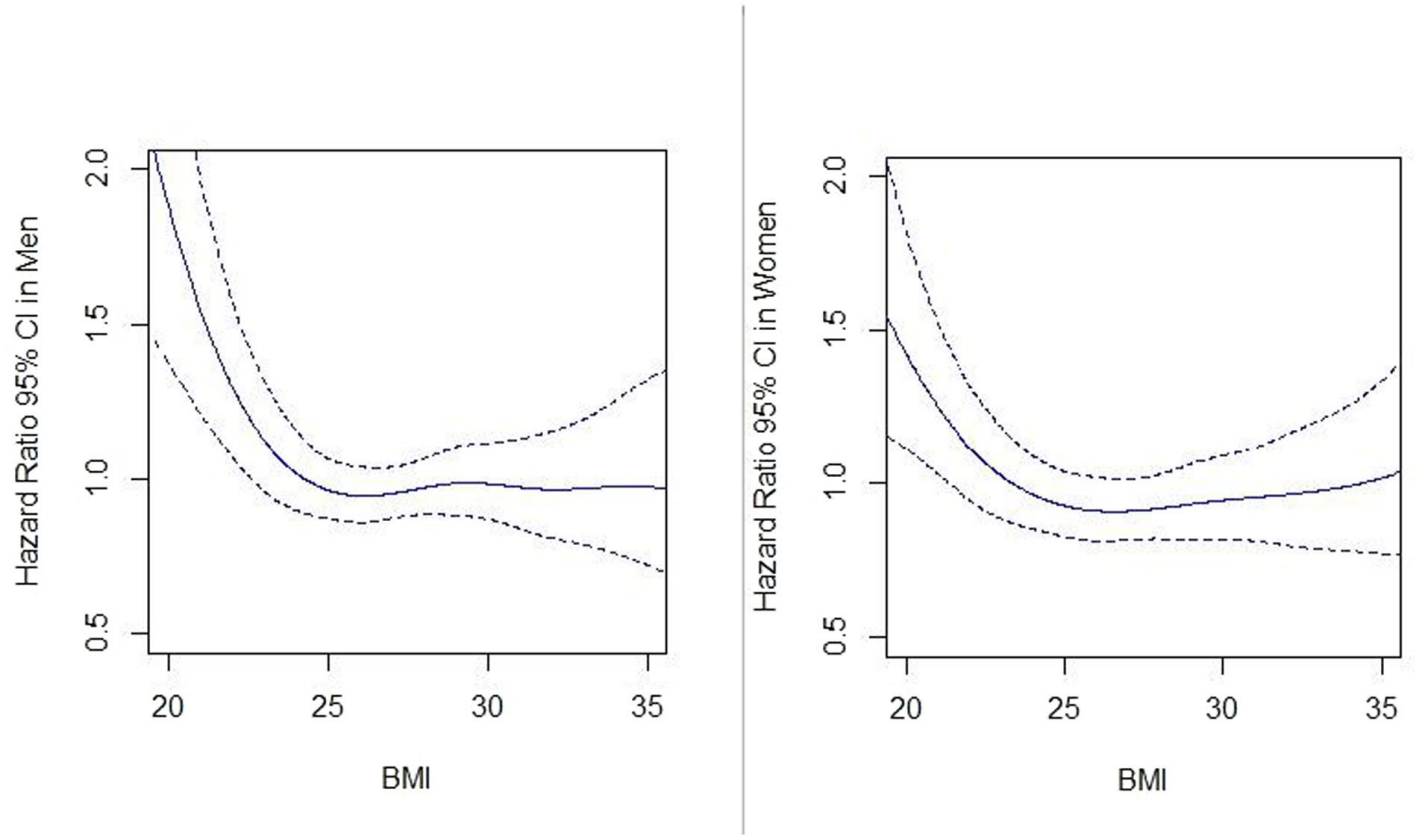
Association between BMI (kg/m^2^) and all-cause mortality in men and women. Hazard ratios of all-cause mortality are calculated by the spline Cox proportional hazard model. Solid lines and dash lines, respectively, represent the hazard ratios and their 95% confidence intervals after adjusting for MM, age, education, family status, smoking habit, alcohol use, and physical activity.

Among men and women aged 65–79 years, MM was positively associated with all-cause mortality (Table 3; Figure 2). The interaction effect between MM and BMI was not significant, however; there were significant differences in survival probabilities of individuals with and without MM at different levels of BMI in men and women. Additionally, participants with higher BMI (BMI >25 kg/m^2^) had a longer survival probability compared to lower BMI (BMI < =25 kg/m^2^) both with and without MM (Figure 3). Based on the spline Cox proportional model, a curvilinear association between BMI and all-cause mortality was specified in men and women. The BMI value related to the highest mortality was for BMI lower than 25 kg/m^2^ (underweight or normal BMI) in both men and women (Figure 4).

**Table 3:** Number of men and women in different age groups stratified by mortality status (alive and died).

Age groups	Men			Women		
	Total	Alive	Died	Total	Alive	Died
65–69	709	645	64	720	666	54
70–74	533	433	100	606	548	58
75–79	413	278	135	408	327	81
80–84	262	136	126	250	151	99
85+	98	28	70	128	52	76
Total	2015	1,520	495	2,112	1744	368

Number of men and women in each age groups who were alive or died until end of follow up (07.10.2016).

### 3.4. Association between multimorbidity and cause-specific mortality

**Figure 5:**
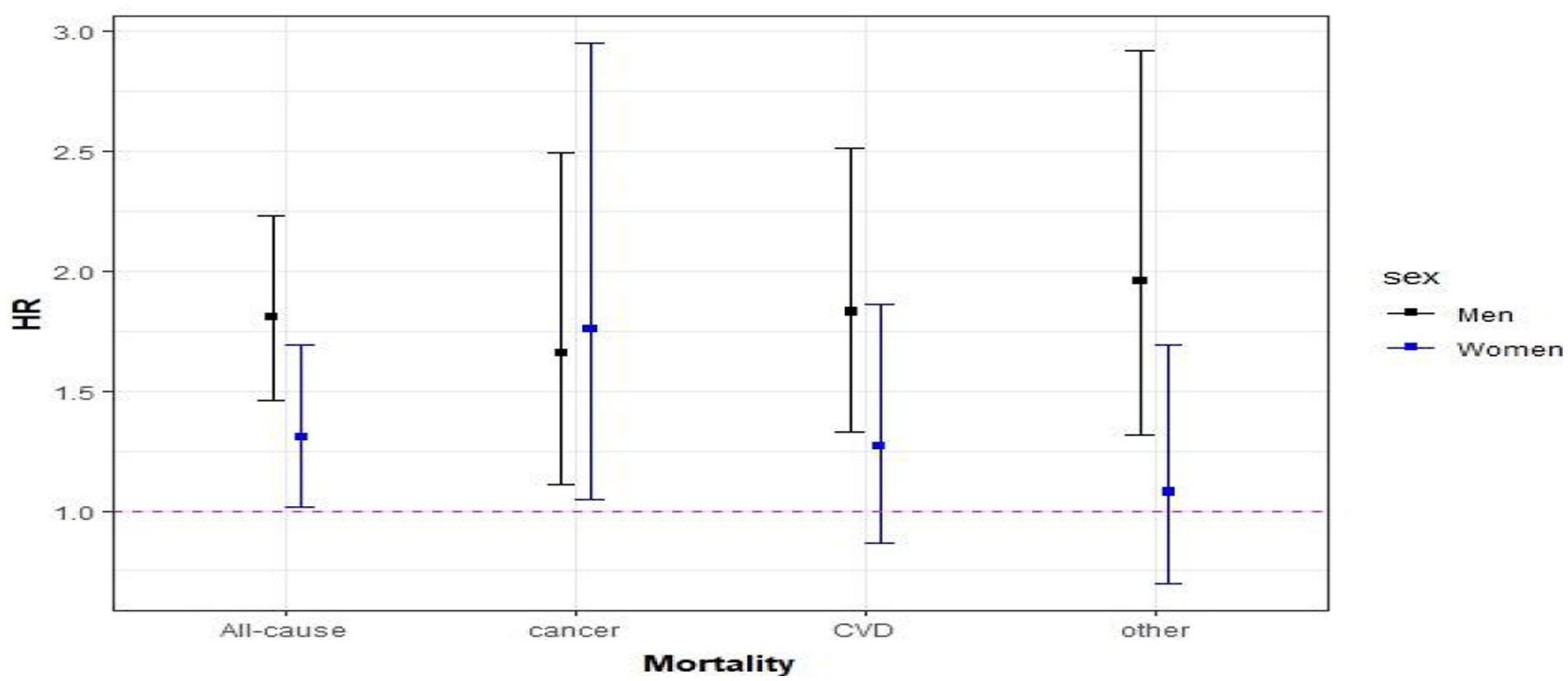
Association between multimorbidity and cause-specific mortality. Hazard ratios (95% CIs) of MM were calculated for all-cause and cause-specific mortality and adjusted for education, family status, smoking habit, alcohol use, BMI, and physical activity.

In the fully adjusted models for cause-specific mortality, the risk of cancer caused mortality was 66% (HR: 1.66, 95% CI: 1.11–2.49) and 76% (HR: 1.76, 95% CI: 1.05–2.95) higher in individuals with MM compared to those without MM in men and women, respectively. In addition, compared with men without MM, the risk of mortality from cardiovascular causes was 83% (HR: 1.83, 95% CI: 1.33–2.51) higher in those who had MM. In women with MM, the hazard ratio for cardiovascular causes was 27% (HR: 1.27, 95% CI: 0.87–1.86) higher than without MM, but the HR was not significantly elevated. For other disease causes, the hazard ratios were (HR: 1.96, 95% CI: 1.32–2.92) and (HR: 1.08, 95% CI: 0.69–1.69) in men and women, respectively, (Figure 5).

### 3.5. Prevalence of single diseases and disease combinations

The prevalence of single and a combination of two and three diseases are shown in Table 4. The five most prevalent paired diseases in men were heart-hypertension, hypertension-eye, diabetes-hypertension, heart-eye, and joint-hypertension. In women, hypertension-eye, heart-hypertension, diabetes-hypertension, joint-hypertension, and heart-eye were the most prevalent pairs.

**Table 4:** Frequency of single disease and the most prevalent combination of two and three diseases and their corresponding adjusted hazard ratios (95% confidence interval) for all-cause mortality stratified by sex.

Frequency (Rank) of single diseases			Combination of two diseases	Men (*n* = 2015)		Women (*n* = 2,112)	
Disease	Men (*n* = 2015)	Women (*n* = 2,112)		Frequency (Rank*)	HR (95% CIs)	Frequency (Rank)	HR (95% CIs)
Hypertension	1,086 (1)	1,206 (1)	Heart-hypertension	381 (1)	1.47 (1.15,1.71)	323 (2)	1.59 (1.17,2.16)
Eye	630 (2)	862 (2)	Hypertension-eye	378 (2)	0.87 (0.78,1.08)	542 (1)	1.25 (0.94,1.67)
Heart	555 (3)	454 (3)	Diabetes-hypertension	268 (3)	1.63 (1.31,2.04)	258 (3)	2.09 (1.52,2.89)
Diabetes	352 (4)	312 (5)	Heart-eye	203 (4)	1.15 (0.91,1.46)	241 (5)	1.52 (1.11,2.11)
Joint	227 (5)	404 (4)	Joint-hypertension	173 (5)	1.15 (0.87,1.51)	247 (4)	1.42 (1.01,2.02)
Lung	207 (6)	200 (7)	Diabetes-eye	146 (6)	1.34 (1.02,1.74)	163 (7)	1.98 (1.38,2.83)
Gastrointestinal	139 (7)	196 (8)	Heart-diabetes	134 (7)	1.87 (1.43,2.45)	108 (12)	2.87 (1.96,4.20)
Stroke	136 (8)	100 (9)	Lung-hypertension	128 (8)	1.34 (0.99,1.81)	134 (8)	1.67 (1.12,2.51)
Cancer	99 (9)	68 (11)	Joint-eye	116 (9)	1.11 (0.81,1.48)	206 (6)	1.12 (0.77–1.62)
Anxiety	90 (10)	223 (6)	Hypertension-anxiety	60 (16)	1.47 (0.95,2.27)	132 (9)	1.64 (1.06,2.55)
Kidney	81 (11)	70 (10)	Joint-heart	91 (12)	1.32 (0.94,1.85)	129 (10)	1.32 (0.86,2.03)
Neurological	60 (12)	50 (12)	**Combination of three diseases**	**Frequency (Rank)**	**HR (95% CIs)**	**Frequency (Rank)**	**HR (95% CIs)**
Liver	41 (13)	47 (14)	Heart-hypertension-eye	145 (1)	1.08 (0.82,1.43)	177 (1)	1.43 (1.00,2.03)
Depression	23 (14)	49 (13)	Heart-diabetes-hypertension	112 (2)	1.81 (1.36,4.42)	91 (4)	2.87 (1.92,4.28)
			Diabetes-hypertension-eye	111 (3)	1.34 (0.99,1.80)	135 (2)	2.61 (1.65,4.13)
			Lung-hypertension-eye	67 (4)	1.14 (0.76,1.77)	80 (6)	1.69 (1.06,2.72)
			Joint-hypertension-eye	67 (5)	1.23 (0.86,1.75)	129 (3)	1.24 (0.81,1.88)
			Joint-heart-hypertension	66 (6)	1.11 (0.73,1.67)	90 (5)	1.52 (0.95,2.43)
			Heart-diabetes-eye	60 (7)	1.69 (1.18,2.41)	63 (9)	2.61 (1.65,4.12)
			Lung-heart-hypertension	53 (8)	1.46 (0.95,2.23)	50 (13)	1.74 (0.98,3.08)
			Joint-diabetes-hypertension	49 (9)	1.61 (1.07,2.39)	51 (12)	2.09 (1.31,3.36)
			Joint-heart-eye	42 (13)	1.09 (0.69,1.72)	73 (7)	1.17 (0.69,1.98)
			Hypertension-eye-anxiety	27 (23)	1.28 (0.69,2.35)	69 (8)	1.66 (0.99,2.76)

*The Rank of disease combination shows the sorted descending rank based on the prevalence of specific combinations among all possible combinations in men and women separately. The heart-hypertension and the hypertension-eye have the highest frequency and first rank for the diseases pair in men and women, respectively. The Hazard ratios (95% CIs) of the specific combination were calculated after adjusting for age, education, family status, smoking habit, alcohol use, BMI, and physical activity. For each hazard ratio, the reference group was considered as individuals who had no or one disease.

### 3.6. Disease combinations and all-cause mortality

The seven most prevalent diseases in men and women (lung, joint, heart, diabetes, hypertension, eye, and anxiety) were selected to check the hazard ratio of the most prevalent combination of two and three diseases. Since the frequencies of quartets and quintets were low, we calculate the HR for the pairs and trios only.

#### 3.6.1. Mortality and combination of two diseases

Men with heart-hypertension, diabetes-hypertension, diabetes-eye, and heart-diabetes had a significantly increased risk of mortality compared with men with one or no disease. Women with heart-hypertension, diabetes-hypertension, heart-eye, joint-hypertension, diabetes-eye, heart-diabetes, lung-hypertension, and hypertension-anxiety had a significantly higher risk of all-cause mortality compared to women with one or no disease. In women, the combination of two diseases resulted in a significantly increased risk of mortality with higher HR value than in men with the same combination (Table 4).

#### 3.6.2. Mortality and combination of three diseases

Men presented significantly higher hazard ratios for individuals who had the combination of heart-diabetes-hypertension, heart-diabetes-eye, and joint-diabetes-hypertension compared with men with one or no disease. However, women showed a significantly higher risk for the combination of heart-hypertension-eye, heart-diabetes-hypertension, diabetes-hypertension-eye, lung-hypertension-eye, heart-diabetes-eye, and joint-diabetes-hypertension (Table 4).

### 3.7. Sensitivity analysis

**Figure 6:**
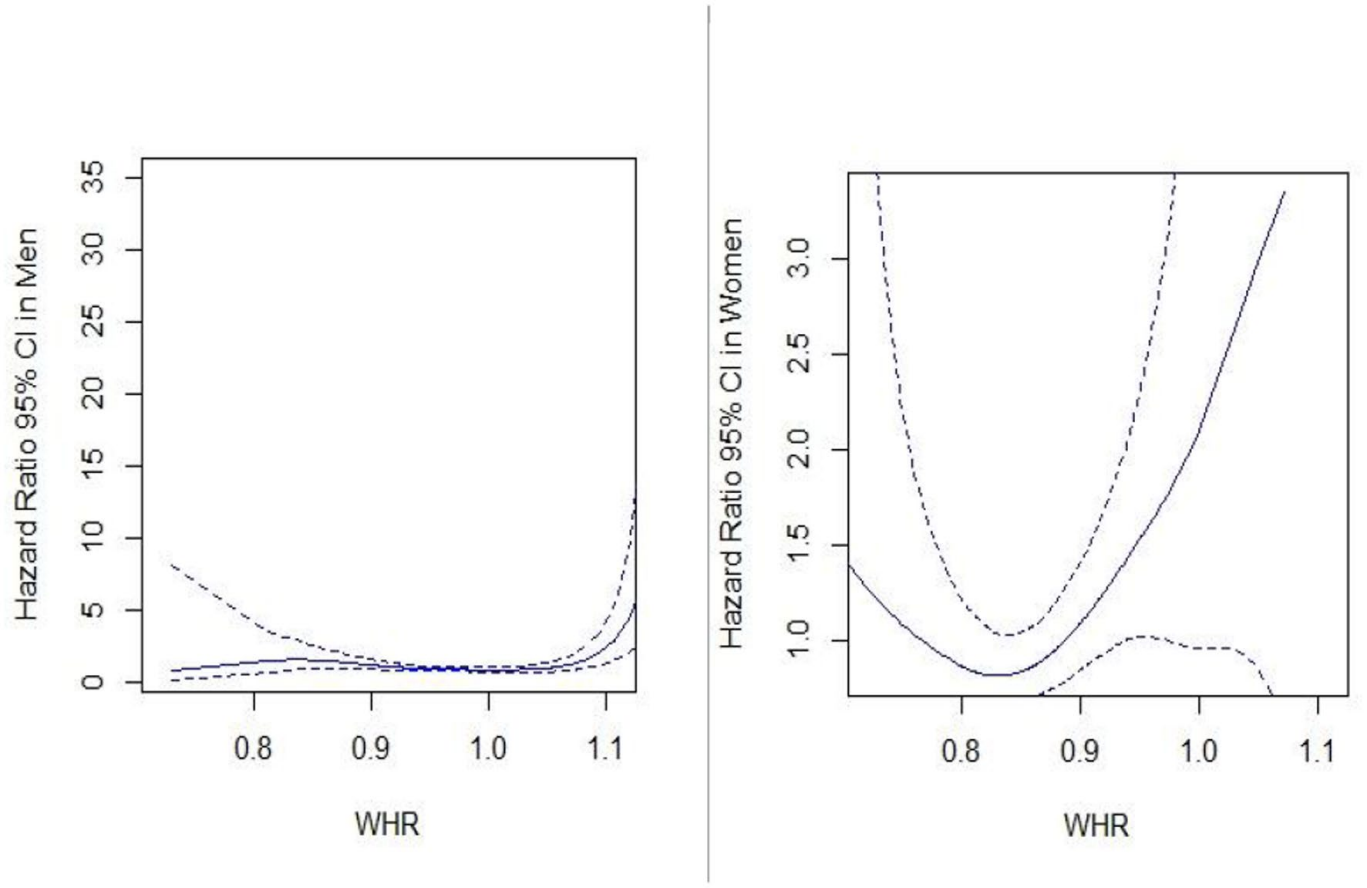
Association between waist-hip ratio (WHR) with all-cause mortality in men and women. Hazard ratios of all-cause mortality are calculated by the spline Cox proportional hazard model. Solid lines and dash lines, respectively, represent the hazard ratios and their 95% confidence intervals after adjusting for MM, age, education, family status, smoking habit, alcohol use, and physical activity.

There is some evidence that the waist-to-hip ratio (WHR) could be a good measure of fat distribution within the body while adjusting for the body shape (20). According to the WHO, a normal WHR range for men is 0.9 or less and 0.85 or less for women, while a WHR of >1.0 can raise the risk of chronic diseases in both male and female. Therefore, we repeated our Cox proportional models using WHR instead of BMI. We only had WHR ratio values for 1,051 participants out of 4,127. Hazard ratios of MM were (HR: 1.89, 95% CI: 1.24–2.78) in men and (HR: 1.12, 95% CI: 0.92–1.69) in women after adjusting the model for WHR instead of BMI. When WHR was used as a continuous variable in the model, a curvilinear (U-shaped) relationship between WHR and all-cause mortality was detected in both men and women (Figure 6). When the cox model was fitted without the MM, the effect estimates for age, particularly in men, increased significantly, but there was no considerable change for the other covariates. Furthermore, only the hazard ratios of MM in men were reduced by roughly 7% after fitting the model without BMI, whereas effect estimates for other covariates did not change significantly (Table 5).

**Table 5:** Association of multimorbidity and covariates with all-cause mortality.

Characteristics		Men			Women		
		Full model	Without MM	Without BMI	Full model	Without MM	Without BMI
Multimorbidity (ref: No)		1.82 (1.48,2.24)	…	1.77 (1.45,2.19)	1.29 (1.01,1.65)	…	1.28 (1.00,1.64)
Age (per year)		1.12 (1.09,1.13)	1.22 (1.14,1.18)	1.12 (1.10,1.13)	1.12 (1.10,1.15)	1.14 (1.11.,1.16)	1.13 (1.11,1.15)
Family status (ref: Living with a partner/spouse)	Living alone, divorced, widowed	1.36 (1.12,1.67)	1.34 (1.10,1.64)	1.27 (0.99,1.61)	1.32 (1.02,1.66)	1.26 (0.99,1.61)	1.27 (0.99,1.61)
Education (ref: 12 years or more)	8–9 years	1.50 (1.09,2.07)	1.46 (1.06,2.01)	1.15 (0.82,1.62)	1.29 (0.88,1.78)	1.16 (0.83,1.64)	1.13 (0.81,1.59)
	10–11 years	1.29 (1.05,1.57)	1.27 (1.04,1.55)	0.93 (0.67,1.28)	1.02 (0.75,1.47)	0.94 (0.68,1.30)	0.93 (0.67,1.28)
Alcohol use (ref: once a week)	Never, rare or former use	1.29 (1.09,2.07)	1.24 (0.89,1.72)	1.25 (0.87,1.79)	1.15 (0.80,1.65)	1.26 (0.88,1.82)	1.27 (0.88,1.82)
	Daily use	1.04 (0.77,1.41)	1.03 (0.76,1.39)	1.11 (0.75,1.65)	1.01 (0.68,1.51)	1.11 (0.75,1.65)	1.13 (0.76,1.67)
Physical activity (ref: active)		1.70 (1.40,2.04)	1.73 (1.43,2.09)	1.66 (1.38,2.00)	1.70 (1.35,2.15)	1.73 (1.37,2.19)	1.69 (1.34,2.14)
BMI (kg/m^2^)		0.97 (0.94,0.99)	0.98 (0.95,1.01)	…	0.99 (0.97,1.01)	0.99 (0.97,1.02)	…
Smoking status (ref: never)	Former smoker	1.16 (0.94,1.43)	1.24 (1.01,1.53)	1.15 (0.93,1.42)	1.08 (0.83,1.39)	1.08 (0.84,1.41)	1.08 (0.83,1.40)
	Current smoker	1.76 (1.21,2.56)	1.81 (1.24,2.62)	1.82 (1.25,2.64)	1.59 (1.03,2.46)	1.61 (1.04,2.48)	1.62 (1.05,2.49)
AIC		6740.871	6773.993	6743.304	5032.326	5034.715	5031.005

Data were represented as hazard ratios with a 95% confidence interval. In sensitivity analyses, MM and BMI were, respectively, omitted from the models.

## 4. Discussion

The KORA-Age study demonstrated a positive association of MM with all-cause mortality. While women had a higher MM prevalence, the HR for the association between MM and all-cause mortality was higher for men than for women. The findings of other studies confirm this finding that women live longer than men but are less healthy (21–24). We could also confirm the finding from a Bavarian Aging Study for individuals older than 65 years old that women with poorer health situations had a lower mortality rate compared to men. They showed that for all ages and morbidity definitions, women had significantly higher life expectancy than men (25).

The sex disparities in MM prevalence and impact of MM on mortality in different parts of the world suggest that there are underlying mechanisms accountable for these differences. One explanation could be that most studies use self-reported information on health status to assess chronic diseases and men prefer to report only severe health conditions. Moreover, women report their chronic diseases and symptoms with more detail and accuracy (26). For instance, a woman suffering from mild angina pectoris might claim that she has a cardiovascular illness, while a man might report it only if he had a heart attack. This reason could also explain the stronger link between MM and CVD mortality among men observed in the present study. Another possible explanation is that the mortality difference between men and women might be influenced by lifestyle factors. Although we performed our analysis after adjusting for socioeconomic factors, the confounder residuals might influence the sex differences in the effect of MM on mortality. While family status was not a significant predictor for women, males who lived alone, divorced, or widowed had a higher significant mortality risk than those who lived with a partner/spouse. Furthermore, males were more likely to use alcohol daily and less likely to be never smokers than females in our study; therefore, sex-specific differences in MM patterns and mortality could be caused by lifestyle differences. Similar results confirmed in self-reported health status that older men with poorer healthy lifestyles had increased mortality risk compared to women (27).

Our research also found that for older men and women, being overweight or obese (BMI > 25 kg/m^2^) is linked to lower mortality risk than having a normal weight (BMI < = 25 kg/m^2^). Although some studies showed that individuals in young or middle age who were overweight (25 < BMI < 30) or had obesity (30 > =BMI) could have an increased risk of mortality compared with normal BMI (18.5 < BMI < =25) adults, there are other studies as well which show a BMI paradox particularly for older adults (aged ≥ 65-year-olds) (24). Moreover, BMI cannot always discriminate between body fat mass and lean tissue properly (28). In sensitivity analysis, we discovered a similar relationship using the waist-to-hip ratio instead of BMI. This paradoxical relationship has been shown in various cohort studies and meta-analyses for those aged over 65 years old. They reported less (29–31) or similar (32) mortality risk for overweight or obese individuals compared to normal weight in older persons. There is still a need to explore the effect of central adiposity on MM and then mortality development in older people. Another possible explanation is that the results pointing at a paradox might mostly be a consequence of misclassification bias, reverse causation, or collider bias (33).

Interestingly, although MM is associated with a higher HR of mortality in men compared to women (based on the general definition of having two or more diseases), the risk of all significant combinations of two and three diseases is higher in women compared to men. In our study, the risk of premature mortality for diseasecombination increased with the number of diagnoses, particularly in women, which is consistent with earlier research (7). Participants with three or four chronic diseases had a 25% higher risk of premature death than those who did not have a chronic disease, and the risk jumped to 80% for those with five or more diseases (34).

According to our findings, hypertension is the most common condition, both alone and in combination, which is consistent with past research (2, 35). Hypertension is strongly associated with risk of premature mortality among individuals with diabetes (36), cardiovascular (37), rheumatoid arthritis (38), and eye disease (39). Men’s mortality risk decreased or remained constant when hypertension was combined with the pairs of diseases; however, there was no specific pattern in women’s risk. Other studies showed that for the old population, hypertension is four times more common in postmenopausal women than in premenopausal women, but only three times more common in age-matched males (40). This sex disparity likely contributes to the lower estrogen level in postmenopausal women since estrogen acts as a protective factor in women (41). Additionally, anxiety and depression in women can increase the risk of hypertension (41). In our study, anxiety appears in the most prevalent combination for women, but these combinations do not show a significantly elevated risk of mortality. Other underlying mechanisms, such as renin-angiotensin, the sympathetic nervous system, the immunological system, lifestyle, and environmental factors could potentially explain sex differences in the presence of hypertension and cardiovascular diseases (42). We also found the heart disease-diabetes combination as the most hazardous in both male and female and it had a higher risk in women compared to men, even when combined with other diseases. Many studies also reported the increased risk of heart failure in the presence of diabetes. This increase might happen because diabetes can raise the risk of atrial fibrillation, and coronary heart disease, which are significant risk factors for heart failure (43). When heart disease-diabetes is combined with hypertension and eye diseases, respectively in trios, the risk decreased in men but remained almost constant in women. In addition, although the joint diseases had the same prevalence in our old men and women, when added into heart-diabetes-hypertension combination, it resulted in significant mortality in women. This finding is in line with other studies that cardiovascular disease is the leading cause of death among rheumatoid arthritis patients, accounting for around 35% of all deaths (44). In contrast to our findings, other studies have found that women suffering from joint disease have less cardiovascular risk compared to men (45, 46). They discovered that, although women are three times more likely than males to have rheumatoid arthritis, they are more protected from coronary artery disease and cardiovascular disease. Different factors such as estrogen level, blood vessel situation, menopausal status, coronary calcium score (46), disability (47) and other lifestyle factors such as alcohol use (48), physical activity (49), BMI (50), and smoking behavior (51) could explain the sex difference risk in diseases combination.

## 5. Strengths and weaknesses

One of the study’s strengths was that it included the most common chronic diseases in the age group 65 and older that might have a significant impact on mortality in older people. Being a very large and informative dataset from the population-based KORA cohort study with older participants and their follow-up information is another strength of our research. We were able to investigate not only the combination of two diseases but also multiple different combinations of diseases due to the large sample size. This database also included data on demographic, sociodemographic, physical, and mental health characteristics, which enabled us to adjust our findings for a variety of MM-related factors.

One potential limitation of this study is that our disease information was collected by questionnaires and telephone interviews, which could result in recall and information biases due to misreporting and non-response in very sick participants. Furthermore, neither the severity of each disease nor geriatric syndromes such as pressure ulcers, incontinence, frailty, falls, functional decline, or delirium were considered in our research. We also did not know which disease occurred first in one individual, which would have allowed for a more exact interpretation. Furthermore, the prevalence of most diseases was relatively low in the KORA-Age population, which could explain the lack of a significant association between some disease combinations and the risk of mortality. Therefore, some of the differences in the strength of associations between MM combinations and mortality between men and women could be explained by the low power induced by the small number of cases in particular combinations of disorders. Furthermore, the data was gathered before the Corona Pandemic and therefore obviously analysis does not take into account the cause-specific mortality by SARS-CoV2.

## 6. Conclusion

The effects of morbidity patterns on mortality in older men and women are highly heterogeneous and depend on the specific disease combinations. Some diseases affected the MM prevalence, but they had no substantial impact on mortality risk. We suggest that future research should look at the morbidity patterns in men and women separately, as they showed different patterns of mortality, which might be due to differences in risk factors profileAlthough hypertension, eye, and joint disease appeared in the most common combinations, these conditions were not as strongly associated with the risk of death as other diseases. In conclusion, MM prevalence itself does not predict mortality but depends on the different disease combination. In the KORA-Age population studied here it was heart disease together with diabetes alone or in combination with other diseases was associated with mortality. Future work should however include geriatric syndromes as well as MM prevalence to add to the understanding what factors contribute to a long and healthy life.

## Data availability statement

The datasets presented in this article are not readily available because there is no participant consent for public data repositories. Requests to access the datasets should be directed to kora.passt@helmholtz-muenchen.de.

## Ethics statement

The studies involving human participants were reviewed and approved by the Ethics Committee of the Bavarian Medical Association (08094). The patients/participants provided their written informed consent to participate in this study.

## Author contributions

AA: conceptualization, methodology, statistical analyses, and original paper draft. AA, AP, BT, and BL: evaluation and interpretation. AA, AP, BT, BL, and MH: revision and editing. AP, BL, BT, MH, and K-HL: main study design, data curation, and quality assurance. AP: supervision. All authors contributed to the article and approved the submitted version.

## Funding

The KORA study was initiated and financed by the Helmholtz Zentrum München – German Research Center for Environmental Health, funded by the German Federal Ministry of Education and Research (BMBF) State Bavaria. Data collection in the KORA study is done in cooperation with the University Hospital of Augsburg. Furthermore, KORA research was supported within the Munich Center of Health Sciences (MC-Health), Ludwig-Maximilian-Universität, as part of LMU innovative. The KORA-Age project was financed by the German Federal Ministry of Education and Research (BMBF FKZ 01ET0713 and 01ET1003A) as part of the health in the old-age program.

## Conflict of interest

The authors declare that the research was conducted in the absence of any commercial or financial relationships that could be construed as a potential conflict of interest.

## Publisher’s note

All claims expressed in this article are solely those of the authors and do not necessarily represent those of their affiliated organizations, or those of the publisher, the editors and the reviewers. Any product that may be evaluated in this article, or claim that may be made by its manufacturer, is not guaranteed or endorsed by the publisher.

## Footnotes

^1^ https://www.r-project.org

## Abbreviations

MM: Multimorbidity
BMI: Body mass index
WHR: Waist to hip ratio
KORA: Cooperative Health Research in the Region of Augsburg
WHO: World Health Organization
IQR: Interquartile range
SD: Standard deviation

## References

[1] Uijen AA van de Lisdonk EH. Multimorbidity in primary care: prevalence and trend over the last 20 years. Eur J Gen Pract. (2008) 14:28–32. doi: 10.1080/13814780802436093 18949641

[2] Kirchberger I Meisinger C Heier M Zimmermann AK Thorand B Autenrieth CS. Patterns of multimorbidity in the aged population. Results from the KORA-age study. PLoS One. (2012) 7:e30556. doi: 10.1371/journal.pone.0030556, PMID: 22291986 PMC3264590

[3] Arshadipour A Thorand B Linkohr B Rospleszcz S Ladwig KH Heier M. Impact of prenatal and childhood adversity effect around world war II on multimorbidity: results from the KORA-age study. BMC Geriatics. (2022) 22:115. doi: 10.1186/s12877-022-02793-2, PMID: 35148691 PMC8832818

[4] Agborsangaya CB Lau D Lahtinen M Cooke T Johnson JA. Health-related quality of life and healthcare utilization in multimorbidity: results of a cross-sectional survey. Qual Life Res. (2013) 22:791–9. doi: 10.1007/s11136-012-0214-7 22684529

[5] Soley-Bori M Bisquera A Ashworth M Wang Y Durbaba S Dodhia H. Identifying multimorbidity clusters with the highest primary care use: 15 years of evidence from a multi-ethnic metropolitan population. Br J Gen Pract. (2021) 72:e190–8. doi: 10.3399/BJGP.2021.0325 PMC8597767 34782317

[6] Quiñones AR Markwardt S Botoseneanu A. Multimorbidity combinations and disability in older adults. J Gerontol. (2016) 71:823–30. doi: 10.1093/gerona/glw035, PMID: 26968451 PMC4888400

[7] Emerging Risk Factors Collaboration Angelantonio ED Kaptoge S Wormser D Willeit P Butterworth AS. Association of Cardiometabolic Multimorbidity with mortality. JAMA. (2015) 314:52–60. doi: 10.1001/jama.2015.7008, PMID: 26151266 PMC4664176

[8] Willadsen TG Siersma V Nicolaisdóttir DR Køster-Rasmussen R Jarbøl DE Reventlow S. Multimorbidity and mortality: a 15-year longitudinal registry-based nationwide Danish population study. J Comorb. (2018) 8:2235042X18804063. doi: 10.1177/2235042X18804063 PMC6194940 30364387

[9] Rizzuto D Melis RJF Angleman S Qiu C Marengoni A. Effect of chronic diseases and multimorbidity on survival and functioning in elderly adults. J Am Geriatr Soc. (2017) 65:1056–60. doi: 10.1111/jgs.14868 28306158

[10] Zheng DD Loewenstein DA Christ SL Feaster DJ Lam BL McCollister KE. Multimorbidity patterns and their relationship to mortality in the US older adult population. PLoS One. (2021) 16:e0245053. doi: 10.1371/journal.pone.0245053, PMID: 33471812 PMC7816983

[11] He K Zhang W Hu X Zhao H Guo B Shi Z. Relationship between multimorbidity, disease cluster and all-cause mortality among older adults: a retrospective cohort analysis. BMC Public Health. (2021) 21:1080. doi: 10.1186/s12889-021-11108-w, PMID: 34090390 PMC8180153

[12] Roman Lay AA Ferreira do Nascimento C Caba Burgos F ADC LH Rivera Zeballos RE Pantoja Silva V. Gender differences between multimorbidity and all-cause mortality among older adults. Curr Gerontol Geriatr Res. (2020) 2020:7816785. doi: 10.1155/2020/7816785 32148480 PMC7049854

[13] Schäfer I Kaduszkiewicz H Nguyen TS van den Bussche H Scherer M Schön G. Multimorbidity patterns and 5-year overall mortality: results from a claims data–based observational study. J Comorb. (2018) 8:2235042X1881658. doi: 10.1177/2235042X18816588, PMID: 30560093 PMC6291890

[14] Peters A Döring A Ladwig KH Meisinger C Linkohr B Autenrieth C. Multimorbidity and successful aging: the population-based KORA-age study. Zeitschrift Gerontol Geriatr. (2011) 44:41–54. doi: 10.1007/s00391-011-0245-7, PMID: 22270973

[15] Chaudhry S Jin L Meltzer D. Use of a self-report-generated Charlson comorbidity index for predicting mortality. Med Care. (2005) 43:607–15. doi: 10.1097/01.mlr.0000163658.65008.ec, PMID: 15908856

[16] Sheikh JI Yesavage JA. A knowledge assessment test for geriatric psychiatry. Hosp Community Psychiatry. (1985) 36:1160–6. PMID: 4065839 10.1176/ps.36.11.1160

[17] Spitzer RL Kroenke K Williams JBW Löwe B. A brief measure for assessing generalized anxiety disorder: the GAD-7. Arch Intern Med. (2006) 166:1092–7. doi: 10.1001/archinte.166.10.1092 16717171

[18] Schederecker F Cecil A Prehn C Nano J Koenig W Adamski J. Sex hormone-binding globulin, androgens and mortality: the KORA-F4 cohort study. Endocr Connect. (2020) 9:326–36. doi: 10.1530/EC-20-0080, PMID: 32168474 PMC7219137

[19] Perna L Mielck A Lacruz ME Emeny RT von Eisenhart Rothe A Meisinger C. The association between resilience and diabetic neuropathy by socioeconomic position: cross-sectional findings from the KORA-age study. J Health Psychol. (2015) 20:1222–8. doi: 10.1177/1359105313510334, PMID: 24287803

[20] Streng KW Voors AA Hillege HL Anker SD Cleland JG Dickstein K. Waist-to-hip ratio and mortality in heart failure. Eur J Heart Fail. (2018) 20:1269–77. doi: 10.1002/ejhf.1244 29963737

[21] Case A Paxson C. Sex differences in morbidity and mortality. Demography. (2005) 42:189–214. doi: 10.1353/dem.2005.0011 15986983

[22] Celli B Vestbo J Jenkins CR Jones PW Ferguson GT Calverley PM. Sex differences in mortality and clinical expressions of patients with chronic obstructive pulmonary disease, the TORCH experience. Am J Respir Crit Care Med. (2011) 183:317–22. doi: 10.1164/rccm.201004-0665OC, PMID: 20813884

[23] Puts MT Lips P Deeg DJ. Sex differences in the risk of frailty for mortality independent of disability and chronic diseases. J Am Geriatr Soc. (2005) 53:40–7. doi: 10.1111/j.1532-5415.2005.53008.x, PMID: 15667374

[24] Deeg DJH Comijs HC Hoogendijk EO van der Noordt M Huisman M. 23-year trends in life expectancy in good and poor physical and cognitive health at age 65 years in the Netherlands, 1993-2016. Am J Public Health. (2018) 108:1652–8. doi: 10.2105/AJPH.2018.304685, PMID: 30359113 PMC6236728

[25] Stephan AJ Schwettmann L Meisinger C Ladwig KH Linkohr B Thorand B. Living longer but less healthy: the female disadvantage in health expectancy. Results from the KORA-age study. Exp Gerontol. (2021) 145:111196. doi: 10.1016/j.exger.2020.111196, PMID: 33310150

[26] Assari S. Gender differences in the predictive role of self-rated health on short-term risk of mortality among older adults. SAGE Open Med. (2016) 4:205031211666697. doi: 10.1177/2050312116666975, PMID: 27651902 PMC5019363

[27] Bowman K Atkins JL Delgado J Kos K Kuchel GA Ble A. Central adiposity and the overweight risk paradox in aging: follow-up of 130,473 UK biobank participants. Am J Clin Nutr. (2017) 106:130–5. doi: 10.3945/ajcn.116.147157, PMID: 28566307 PMC5486197

[28] Tchernof A Després JP. Pathophysiology of human visceral obesity: an update. Physiol Rev. (2013) 93:359–404. doi: 10.1152/physrev.00033.2011 23303913

[29] Flegal KM Kit BK Orpana H Graubard BI. Association of all-cause mortality with overweight and obesity using standard body mass index categories: a systematic review and meta-analysis. JAMA. (2013) 309:71–82. doi: 10.1001/jama.2012.113905, PMID: 23280227 PMC4855514

[30] Winter JE MacInnis RJ Wattanapenpaiboon N Nowson CA. BMI and all-cause mortality in older adults: a meta-analysis. Am J Clin Nutr. (2014) 99:875–90. doi: 10.3945/ajcn.113.068122 24452240

[31] Bosello O Vanzo A. Obesity paradox and aging. Eat Weight Disord. (2021) 26:27–35. doi: 10.1007/s40519-019-00815-4 31865598

[32] Pischon T Boeing H Hoffmann K Bergmann M Schulze MB Overvad K. General and abdominal adiposity and risk of death in Europe. N Engl J Med. (2008) 395:2105–20. doi: 10.1056/NEJMoa0801891 19005195

[33] Banack H Stokes A. The obesity paradox may not be a paradox at all. Int J Obes. (2017) 41:1162–3. doi: 10.1038/ijo.2017.99 28584251

[34] Caughey GE Ramsay EN Vitry AI Gilbert AL Luszcz MA Ryan P. Comorbid chronic diseases, discordant impact on mortality in older people: a 14-year longitudinal population study. J Epidemiol Community Health. (2010) 64:1036–42. doi: 10.1136/jech.2009.088260, PMID: 19854745

[35] Jacob L Breuer J Kostev K. Prevalence of chronic diseases among older patients in German general practices. Ger Med Sci. (2016) 14:Doc03. doi: 10.3205/000230 26977142 PMC4779902

[36] Charoensri S Kritmetapak K Tangpattanasiri T Pongchaiyakul C. The impact of new-onset diabetes mellitus and hypertension on all-cause mortality in an apparently healthy population: a ten-year follow-up study. J Diabetes Res. (2021) 9:1–7. doi: 10.1155/2021/3964013 PMC8589482 34778463

[37] Redon J Tellez-Plaza M Orozco-Beltran D Gil-Guillen V Pita Fernandez S Navarro-Pérez J. Impact of hypertension on mortality and cardiovascular disease burden in patients with cardiovascular risk factors from a general practice setting: the ESCARVAL-risk study. J Hypertens. (2016) 34:1075–83. doi: 10.1097/HJH.0000000000000930, PMID: 27074896

[38] Baghdadi LR Woodman RJ Shanahan EM Mangoni AA. The impact of traditional cardiovascular risk factors on cardiovascular outcomes in patients with rheumatoid arthritis: a systematic review and meta-analysis. PLoS One. (2015) 10:e0117952. doi: 10.1371/journal.pone.0117952, PMID: 25689371 PMC4331556

[39] Konstantinidis L Guex-Crosier Y. Hypertension and the eye. Curr Opin Ophthalmol. (2016) 27:514–21. doi: 10.1097/ICU.0000000000000307 27662019

[40] Benjamin EJ Blaha MJ Chiuve SE Cushman M das S Deo R. Heart disease and stroke Statistics-2017 update: a report from the American Heart Association. Circulation. (2017) 135:e146–603. doi: 10.1161/CIR.0000000000000485, PMID: 28122885 PMC5408160

[41] Lima R Wofford M Reckelhoff JF. Hypertension in postmenopausal women. Curr Hypertens Rep. (2012) 14:254–60. doi: 10.1007/s11906-012-0260-0, PMID: 22427070 PMC3391725

[42] Colafella KMM. Sex-specific differences in hypertension and associated cardiovascular disease. Nat Rev Nephrol. (2018) 14:185–201. doi: 10.1038/nrneph.2017.189 29380817

[43] Aune D Schlesinger S Neuenschwander M Feng T Janszky I Norat T. Diabetes mellitus, blood glucose and the risk of heart failure: a systematic review and meta-analysis of prospective studies. Nutr Metab Cardiovasc Dis. (2018) 28:1081–91. doi: 10.1016/j.numecd.2018.07.005, PMID: 30318112

[44] Ometto F Fedeli U Schievano E Botsios C Punzi L Corti MC. Cause-specific mortality in a large population-based cohort of patients with rheumatoid arthritis in Italy. Clin Exp Rheumatol. (2018) 36:636–42. PMID: 29533757

[45] Rohrich DC van de Wetering EHM Rennings AJ Arts EE Meek IL den Broeder AA. Younger age and female gender are determinants of underestimated cardiovascular risk in rheumatoid arthritis patients: a prospective cohort study. Arthritis Res Ther. (2021) 23:2. doi: 10.1186/s13075-020-02384-9, PMID: 33397472 PMC7784252

[46] Adawi M Gurovich B Firas S Watad A Bragazzi N Amital H. Gender differences in cardiovascular risk of patients with rheumatoid arthritis. QJM. (2019) 112:657–61. doi: 10.1093/qjmed/hcz124 31147698

[47] Wang Z Peng W Li M Li X Yang T Li C. Association between multimorbidity patterns and disability among older people covered by long-term care insurance in Shanghai, China. BMC Public Health. (2021) 21:418. doi: 10.1186/s12889-021-10463-y 33639902 PMC7912511

[48] Ceylan-Isik AF McBride SM Ren J. Sex difference in alcoholism: who is at a greater risk for development of alcoholic complication. Life Sci. (2010) 87:133–8. doi: 10.1016/j.lfs.2010.06.002, PMID: 20598716 PMC2913110

[49] Ge L Yap CW Heng BH. Sex differences in associations between multimorbidity and physical function domains among community-dwelling adults in Singapore. PLoS One. (2018) 13:e0197443. doi: 10.1371/journal.pone.0197443, PMID: 29758072 PMC5951575

[50] Booth HP Prevost AT Gulliford MC. Impact of body mass index on the prevalence of multimorbidity in primary care:cohort study. Fam Pract. (2014) 31:38–43. doi: 10.1093/fampra/cmt061, PMID: 24132593 PMC3902211

[51] Kim J Lee M Dan H. Gender differences in factors affecting life satisfaction of the elderly with multimorbidity in Korea. Nurs Rep. (2021) 11:54–63. doi: 10.3390/nursrep11010006, PMID: 34968312 PMC8608087

